# Pancreatic enzyme supplementation versus placebo for improvement of gastrointestinal symptoms in non-responsive celiac disease: A cross-over randomized controlled trial

**DOI:** 10.3389/fmed.2022.1001879

**Published:** 2023-01-04

**Authors:** Shakira Yoosuf, Caitlin G. Barrett, Konstantinos Papamichael, Sarah E. Madoff, Satya Kurada, Joshua Hansen, Jocelyn A. Silvester, Amelie Therrien, Prashant Singh, Melinda Dennis, Daniel A. Leffler, Ciaran P. Kelly

**Affiliations:** ^1^Celiac Center, Division of Gastroenterology, Department of Medicine, Beth Israel Deaconess Medical Center, Boston, MA, United States; ^2^Celiac Research Program, Harvard Medical School, Boston, MA, United States; ^3^Chettinad Hospital and Research Institute, Chennai, India; ^4^Maimonides Medical Center, Brooklyn, NY, United States; ^5^Division of Gastroenterology and Hepatology, Indiana University School of Medicine, Indianapolis, IN, United States; ^6^Division of Gastroenterology, Hepatology and Nutrition, Boston Children’s Hospital, Boston, MA, United States; ^7^Division of Gastroenterology, University of Michigan, Ann Arbor, MI, United States; ^8^Takeda Pharmaceuticals International Co., Cambridge, MA, United States

**Keywords:** diarrhea, sprue, malabsorption, exocrine pancreatic insufficiency, proton pump inhibitors, pancreas, dyspepsia

## Abstract

**Background:**

Pancreatic Exocrine Insufficiency (PEI) is a possible cause of recurrent/persistent symptoms in celiac disease. Although pancreatic enzyme supplementation may be used to treat non-responsive celiac disease (NRCD) in clinical practice, clinical outcomes are variable and there is limited and low quality evidence to support this practice. The aim of this study was to assess the efficacy of pancreatic enzyme supplements (PES) for improvement of gastrointestinal symptoms in NRCD.

**Methods:**

Prospective, randomized, placebo-controlled, double-blind, cross-over trial in adults with NRCD examining Celiac Disease-Gastrointestinal Symptom Rating Scale (CeD-GSRS) scores on PES (pancrelipase co-administered with omeprazole) versus placebo (omeprazole only) during a 10-day treatment period. The study was registered under the clinical trials registry (https://clinicaltrials.gov/ number, NCT02475369) on 18 Jun 2015.

**Results:**

Twelve participants (nine female) were included in the per-protocol analysis; one participant had low fecal elastase-1. Pancrelipase was not associated with significant change in CeD-GSRS compared to placebo (−0.03 versus −0.26; *P* = 0.366). There was a significant decrease in mean values of total CeD-GSRS scores (3.58 versus 2.90, *P* = 0.004), abdominal pain (2.92 versus 2.42, *P* = 0.009), and diarrhea sub-scores (3.44 versus 2.92, *P* = 0.037) during the run-in period with omeprazole.

**Conclusion:**

In this prospective, cross-over randomized, placebo-controlled study, PES did not improve symptoms in patients with NRCD. It is unclear whether this is a trial effect or related to administration of omeprazole.

## Introduction

Non-responsive celiac disease (NRCD) is characterized by persistent/recurrent symptoms of celiac disease (CeD) despite 6–12 months of adherence to a gluten-free diet (GFD) (1). Pancreatic Exocrine Insufficiency (PEI) is one of the suspected contributory factors for these symptoms, occurring in an estimated 12% of NRCD patients (2). Multiple mechanisms have been proposed to account for PEI in CeD, including enterokinase deficiency secondary to villous atrophy which may impair activation of pancreatic enzymes (3, 4). Although pancreatic enzyme supplementation may be used to treat NRCD in clinical practice, results are variable and there is limited and low quality evidence to support this practice.

Based on symptomatic response to a trial of pancreatic enzyme supplements (PES), the prevalence of PEI has been found to be 10–18% in NRCD patients in retrospective chart reviews (5, 6). Further, one previous clinical trial showed PES to be useful in alleviating persistent symptoms in NRCD, but exclusively included patients with low fecal elastase-1 levels, and studied their diarrheal symptoms only (7). While earlier reports suggested that loss of >90% pancreatic function (severe PEI) is required to cause clinically significant diarrhea, more recent studies point to symptoms occurring in mild-moderate PEI (8), which may not necessarily be accompanied by low fecal elastase-1 levels (9). Therefore, we examined the efficacy of PES in patients with a broader range of NRCD symptoms, such as abdominal pain and bloating, irrespective of the presence of clinically demonstrable pancreatic dysfunction as assessed by fecal elastase-1 levels.

## Materials and methods

**FIGURE 1:**
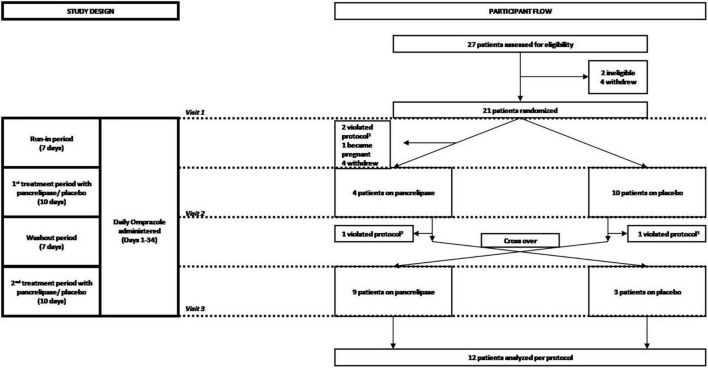
Consort diagram of the randomization and follow-up of study participants. Participants were screened for eligibility, assessed for severity of celiac disease symptoms and randomized at visit 1, which corresponded to the beginning of a 7 day run-in period during which participants received 20 mg omeprazole orally. Omeprazole was also administered during the first 10-day period of treatment with either pancreatic enzyme supplements (pancrelipase) or placebo, a 7 day wash-out (starting with visit 2) and the second 10-day treatment with pancreatic enzyme supplements or placebo (starting with visit 3). Participants who completed the overall 34-day course were then analyzed per-protocol. ^1^Two patients failed run-in; ^2^one patient did not complete data collection; ^3^one patient started placebo before washout.

We conducted a prospective, randomized, double-blind, placebo-controlled, cross-over trial to determine the efficacy of PES for treatment of gastrointestinal (GI) symptoms in NRCD patients. Subjects with NRCD on a strictly GFD recruited at Beth Israel Deaconess Medical Center (BIDMC) between July 2015 and April 2017 were randomized 1:1 to initial treatment with either pancrelipase (Viokace^®^, Confab Laboratories, St-Hubert, QC, Canada) coadministered with omeprazole, or placebo with omeprazole (Figure 1). Coadministration of omeprazole with Viokace^®^ is recommended in order to prevent gastric inactivation of these non-enteric coated PES. Therefore, we had a 1-week run-in period to ensure omeprazole was therapeutic prior to initiation of pancreatic enzyme supplementation. Inclusion criteria were: biopsy-confirmed CeD; age 18–80 years; self-reported strict adherence to GFD as assessed by an expert dietitian and the Celiac Dietary Adherence Test (CDAT) (10); and persistent GI symptoms (Celiac Disease-Gastrointestinal Symptom Rating Scale or CeD-GSRS score ≥3 in the highest domain at baseline visit) (11). Those with pork allergy, limited English proficiency, lactose intolerance, pregnancy, history of chronic active GI disease (other than CeD), major GI surgeries or other conditions that would interfere with subjects’ participation or confound study results were excluded.

Subjects were screened and randomized at visit 1, which corresponded to the beginning of a 7-day run-in period during which participants received omeprazole 20 mg orally. Omeprazole administration was continued through to the end of the study, including the first 10-day period of treatment with either PES (pancrelipase) or placebo, followed by a 7-day wash-out and the second 10-day treatment with pancrelipase or placebo (Figure 1). Participants who completed the 34-day study were analyzed per-protocol. Fecal elastase-1 and celiac serology levels were determined at the baseline visit. There were two additional visits at the end of each treatment period. At all three visits, medication logs were checked and symptoms were assessed with the Celiac Symptom Index (CSI; modified to have a 1-week recall period, from the original 4-week recall) and the CeD-GSRS (total score as well as scores in three domains – abdominal pain, indigestion, and diarrhea) (12).

Participants were randomized in a 1:1 ratio using a computer-generated sequence in blocks of four. The hospital pharmacy group performed the randomization and dispensed the study drug with no further involvement in the study. To maintain allocation concealment, identical, sequentially numbered containers were used for treatment and placebo. During the treatment phase, participants received Viokace, a porcine derived pancrelipase that contains lipase, amylase, and protease administered with meals and snacks. Allocation concealment was maintained using equal numbers of visually indistinguishable capsules for placebo and active treatment. Each capsule of Viokace contained 20,880 USP units of lipase, 78,300 USP units of protease and 78,300 USP units of amylase and the number of capsules was based upon weight: >59 kg: 6/meal, 4/snack; 53–59 kg: 5/meal, 4/snack; 47–52 kg: 5/meal, 3/snack; and 40–46 kg: 4/meal, 3/snack.

The primary outcome was change in mean CeD-GSRS on PES versus placebo. Secondary outcomes included change in mean CSI, and CeD-GSRS individual domain scores (abdominal pain, indigestion, and diarrhea) on PES versus placebo and correlation of these outcomes with baseline fecal elastase-1. For sample size calculation, we hypothesized a mean difference in CeD-GSRS of 0.4 between treatment and placebo (11). Assuming SD = 0.55, we needed 32 patients to achieve 80% power at α = 0.05. The primary outcome was evaluated using Wilcoxon signed rank test and linear mixed effects model using the nlme package (13). In the model, treatment (PES or placebo), time (before or after treatment), treatment × time interaction, sequence (PES first or placebo first), period (before or after the crossover), age and sex were considered fixed effects and intercepts for subjects were random effects. Additionally, proportions of patients with >30 and >50% reductions in CeD-GSRS total and abdominal pain scores were compared between the two interventions using McNemar’s test. All statistical tests were performed with R (R Core Team, 3.6.1). A two-sided *P*-value < 0.05 was considered statistically significant. The study was approved by the BIDMC Institutional Review Board; ClinicalTrials.gov number, NCT02475369. Informed consent was obtained from all individual participants included in the study.

## Results

We enrolled and randomized 21 patients (17 female, median age: 41 years, range: 23–70 years). Initial treatment randomizations were 11 PES and 10 placebo (Figure 1). The trial was terminated prematurely by the sponsor for administrative reasons. Due to dropouts, a per-protocol analysis was performed on the 12 participants that completed the study (9 female; 3 initial treatment placebo) (Table 1). None of the eight dropouts in the initial PES arm were related to adverse events (AE). Also, dropouts had similar baseline characteristics. Treatment compliance, as assessed by pill count, was >90% on PES and >70% on placebo, with no severe AE reported.

**TABLE 1:** Baseline characteristics of patients.

	Population at baseline			Population included in per-protocol analysis		
Characteristic	All patients (*N* = 21)^¥^	First intervention pancreatic enzyme supplement (*N* = 11)^¥^	First intervention placebo (*N* = 10)^¥^	All patients (*N* = 12)*	First intervention pancreatic enzyme supplement (*N* = 3)*	First intervention placebo (*N* = 9)*
Female (%)	17 (81)	7 (64)	10 (100)	9 (75)	1 (33)	8 (89)
Age, years, median (range)	41 (23–70)	30 (23–69)	46 (24–70)	42 (24–70)	30 (26–41)	45 (24–70)
White race (%)	20 (95)	11 (100)	9 (90)	11 (92)	3 (100)	8 (90)
BMI^a^, kg/m^2^, median (range)	23.9 (17.2–35.5)	22.8 (17.2–29.7)	28.4 (18.2–35.5)	28.4 (18.2–35.5)	22.3 (21.5–29.7)	28.9 (18.2–35.5)
Months since diagnosis	69 (16–187)	55 (16–137)	111 (16–187)	79.6 (16–187)	69 (54–137)	90 (16–187.5)
Months on a gluten-free diet	69 (16–191)	55 (17–111)	111 (16–187)	79 (16–187)	69 (54–137)	90 (16–187)
Months with symptoms	24 (6–416)	229 (6–249)	17 (16–70)	24 (5–250)	229 (6–250)	17 (16–70)
Baseline CeD-GSRS total score, median (range)	2.9 (1.2–4.6)	3.4 (1.2–4.6)	2.8 (1.4–4.3)	2.9 (1.4–4.4)	3.7 (1.4 –4.4)	2.8 (1.4–4.3)
Baseline CeD-GSRS abdominal pain subdomain score, median (range)	1 (0.67–2)	1.33 (0.67–1.67)	1 (0.67–2)	1.17 (0.67–2)	1.33 (0.67–1.67)	1 (0.67–2)
Baseline indigestion CeD-GSRS^c^ score, median (range)	3.4 (1.7–6)	3.2 (1.8–6.0)	3.5 (2.8–4.8)	3.5 (2.3–5.5)	3.25 (2.3–5.5)	3.5 (2.8–4.8)
Baseline diarrhea CeD-GSRS^c^ score, median (range)	3.1 (1.0–6.3)	3.3 (1–6.3)	3 (1.3–5.3)	3.1 (1.3–5.3)	5.0 (1.3–5.3)	3.0 (1.3–5.3)
Baseline CSI^b^ score, median (range)	34.5 (26–57)	31 (26–46)	35 (32–57)	35 (26–57)	41 (26–41)	35 (32–57)
Baseline CDAT^d^ score, median (range)	12 (7–16)	9 (7–14)	14 (9–16)	13 (7–16)	9 (7–9)	14 (9–16)
tTG-IgA^e^, median (range)	10 (2–28)	5 (2–28)	13 (2–27)	12 (2–27)	5 (3–13)	13 (2–27)

^¥^Patients screened at baseline. *Patients included in per-protocol analysis. *P*-value not significant for all intergroup comparisons. ^a^Body mass index. ^b^Celiac Symptom Index: scores range from 16 to 80, with greater scores indicating worse symptoms. ^c^Celiac Disease Gastrointestinal Symptom Response Scale: scores range from 1 to 7, with greater scores indicating worse symptoms. It has three domains: abdominal pain, indigestion, and diarrhea with each domain having a score ranging from 1 to 7 and greater scores indicating worse symptoms. ^d^Celiac Dietary Adherence Test: scores range from 5 to 35, with greater scores indicating poorer adherence. ^e^IgA anti-human tissue transglutaminase assay (Inova Diagnostics, Inc., San Diego, CA, USA): negative <20, borderline 20–30, positive >30.

Celiac Disease-Gastrointestinal Symptom Rating Scale total (3.58 versus 2.90, *P* = 0.004), and abdominal pain scores (2.92 versus 2.42, *P* = 0.009) were significantly reduced following the 7-day run-in period. Comparing pre- and post-intervention time-points, no significant changes were found in CeD-GSRS or CSI on PES compared with placebo by the Wilcoxon method and mixed effects model (Table 2). However, there was a statistically significant period effect associated with improvement in the CeD-GSRS total (*P* = 0.04) and indigestion domain (*P* = 0.008) scores.

**TABLE 2:** Symptom response to treatment with pancreatic enzyme supplement compared to placebo.

Outcome	Results by study phase, mean (SD)							Difference of treatment effect from the linear mixed effects model^b^	
	Pancreatic enzyme supplement			Placebo			*P*-value^a^	Mean (SE)	*P*-value
CeD-GSRS score	Before	After	Delta	Before	After	Delta			
Total	2.57 (1.04)	2.55 (0.89)	−0.03 (0.30)	2.78 (0.94)	2.54 (1.06)	−0.26 (0.59)	0.366	0.16 (0.19)	0.407
Abdominal pain domain	2.08 (0.98)	2.11 (0.94)	0.03 (0.26)	2.44 (0.81)	2.17 (1.08)	−0.28 (0.53)	0.133	0.01 (0.13)	0.918
Indigestion domain	2.79 (1.08)	2.71 (0.89)	−0.08 (0.46)	3.02 (0.93)	2.85 (1.02)	−0.17 (0.81)	1	0.13 (0.26)	0.628
Diarrhea domain	2.78 (1.57)	2.78 (1.62)	0 (0.70)	2.86 (1.64)	2.5 (1.56)	−0.36 (1.16)	0.359	0.34 (0.38)	0.378
CSI score	34.4 (10.3)	34.90 (9.5)	−1.5 (4.50)	39.2 (10.1)	36.1 (11.8)	3.08 (4.98)	0.052	−0.29 (1.95)	0.882

^a^Comparisons of the within-pair differences (delta) in symptom scores after pancreatic enzyme supplement and placebo treatment, by the Wilcoxon signed rank test. ^b^Estimated difference of the effect of pancreatic enzyme supplement versus placebo by the linear mixed effects model using the nlme package. Sequence effect or the carry-over effect was not significant for the CeD-GSRS total score domain (*P* = 0.807) and its subdomains as well as for the CSI score (*P* = 0.768).

No subject on PES and three (25%) on placebo had a >30% reduction in both total CeD-GSRS and the abdominal pain domain score compared to the end of run-in period (*P* = 0.008), with none having >50% reduction. Only one subject had low fecal elastase-1 at baseline (8%, 95% CI: 1.5–35%). Five subjects (42%) had fecal elastase-1 between 200 and 500 μg/g stool and six subjects (50%) had values >500 μg/g stool. Baseline fecal elastase-1 did not correlate significantly with differences in total CeD-GSRS score on PES versus placebo (*R* = 0.34, *P* = 0.280).

## Discussion

This is the first prospective, controlled trial to examine the efficacy of PES in NRCD. PES was not associated with significant symptom improvement as assessed by CeD-GSRS or CSI. However, CeD-GSRS decreased significantly during the run-in period during which omeprazole was administered. Thus, it is unclear whether improvement is attributable to the well-documented trial effect in CeD whereby enrollment in a trial leads to symptomatic improvement, presumably due to stricter GFD adherence (11, 14) or if there is a beneficial effect of omeprazole. The same factors could also explain the significant period effect associated with improvement of CeD-GSRS total and indigestion domain scores after the run-in period, even though overall, the improvement in these scores was not significant in the mixed effects model. Literature on the role of proton pump inhibitors (PPIs) in NRCD is scant, but a significant proportion of our cohort had PPI-responsive symptoms such as epigastric pain, which is known to occur frequently in NRCD (1).

Our cohort had one subject (8%, 95% CI: 1.5–35%) with low fecal elastase-1 levels, which is consistent with prior studies of NRCD (5, 15). Thus, it did not include exclusively individuals with known PEI, unlike a previous uncontrolled open-label trial that demonstrated significant reduction of diarrhea in 18 out of 20 NRCD subjects, all having low fecal elastase-1 levels (7). Further, our cohort was more representative of the general NRCD population given that we recruited patients with a wide variety of symptoms including abdominal pain and bloating, and not specifically diarrhea. Similarly, the baseline CeD-GSRS and CSI scores are similar to those from other general NRCD cohorts recruited in previous studies (11, 16). Compliance to PES was adequate with weight-based dosing with meals and/or snacks and co-administration of omeprazole, as is recommended for non-enteric coated pancreatic enzymes (17). The normal fecal elastase-1 levels and lack of response to PES in our subjects indicate that most symptoms were unlikely to be attributable to PEI. This is consistent with a previous retrospective study at our center, showing an absence of response to PES in the NRCD population (18). Furthermore, the only patient with low fecal elastase-1 in our study failed to show resolution of diarrhea with PES, but fecal elastase-1 may have been artificially low in the liquid stool sample (19).

This study attempted to address the knowledge gap concerning the use of PES for NRCD irrespective of the presence of demonstrable PEI, which is a clinically relevant question. Limited data from previous retrospective studies have documented symptomatic improvement with trials of PES in 10–18% of NRCD patients (5, 6). However, with a rigorous study design, validated measures of GI symptoms and GFD adherence and standardized weight-based dosing of pancreatic enzymes, our trial showed contrary results. Study limitations include lack of a run-in period prior to omeprazole and trial termination before reaching an adequate sample size, with potential for a type 2 error. Also, despite randomization, protocol violations resulted in an unbalanced distribution in the two initial treatment groups (nine subjects assigned to placebo and three to pancrelipase); however, that did not significantly alter the relative distribution of baseline characteristics between the groups. Despite the small sample size, most patients showed no improvement on PES, which indicates that PEI may not be a common contributor to persistent symptoms in an unselected NRCD population.

Although empiric trials of PES have been suggested, this should not be routine until larger trials with robust study designs similar to ours demonstrate efficacy. As well, future studies may benefit from incorporation of recently available fecal and urine tests for gluten immunogenic peptides to select or stratify the population based upon ongoing gluten exposure, and provide objective information to control for a potential trial effect related to improved GFD adherence/reduced gluten exposure. Although representative of the NRCD population, the degree of symptoms of patients in our study was mild-moderate, which reduced the ability to detect a small improvement. To further characterize the effectiveness of PES, future prospective studies should examine larger NRCD cohorts with varying fecal elastase levels and with a wider range of symptom severity.

## Conclusion

In this prospective cross-over randomized controlled trial of PES versus placebo, patients showed no significant improvement on PES, which indicates that PEI may not be a common contributor to persistent symptoms in NRCD population.

## Data availability statement

The raw data supporting the conclusions of this article will be made available by the authors, without undue reservation.

## Ethics statement

The studies involving human participants were reviewed and approved by the Beth Israel Deaconess Medical Center Committee on Clinical Investigation. The patients/participants provided their written informed consent to participate in this study.

## Author contributions

CK and DL: study concept, design, and supervision. CB, CK, DL, JS, JH, MD, PS, SK, SM, and SY: acquisition of patient data. CK, DL, JS, KP, PS, and SY: statistical analysis. AT, CB, CK, DL, JS, KP, MD, PS, and SY: drafting of the manuscript, data interpretation, and critical revision of the manuscript for important intellectual content. All authors contributed to the article and approved the submitted version.
